# Using menopausal hormone therapy after a cancer diagnosis in Ireland

**DOI:** 10.1007/s11845-022-02947-6

**Published:** 2022-02-09

**Authors:** Fionán Donohoe, Yvonne O’Meara, Aidin Roberts, Louise Comerford, Catherine M. Kelly, Janice M. Walshe, Deirdre Lundy, Martha Hickey, Donal J. Brennan

**Affiliations:** 1 Living Well Cancer Programme, UCD Gynaecological Oncology Group, UCD School of Medicine, Catherine McAuley Research Centre, Mater Misericordiae University Hospital, Eccles Street, Dublin 7, Ireland; 2 Dept. of Medical Oncology, Mater Misericordiae University Hospital, Eccles Street, Dublin 7, Ireland; 3 Dept. of Medical Oncology, St. Vincent’s University Hospital, Elm Park, Dublin 4, Ireland; 4 Reproductive and Sexual Health Co-Ordinator, Irish College of General Practitioners, Lincoln Place, Dublin 2, Ireland; 5 Dept. of Obstetrics and Gynaecology, University of Melbourne, Royal Women’s Hospital, Melbourne, VIC Australia; 6 UCD Gynaecological Oncology Group, UCD School of Medicine, Catherine McAuley Research Centre, Mater Misericordiae University Hospital, Belfield, Dublin 4, Ireland

**Keywords:** Cancer, Menopause, Survivorship

## Abstract

**Background:**

Menopause may cause a constellation of symptoms that affect quality of life. Many women will have menopause induced or exacerbated by treatment for cancer whether that be through surgery, chemotherapy, radiotherapy, or anti-endocrine therapy. As treatments advance, the number of people living with and beyond a cancer diagnosis is set to increase over the coming years meaning more people will be dealing with the after effects of cancer and its treatment.

**Aims:**

This review aims to summarise available data to guide clinicians treating women with menopausal symptoms after the common cancer diagnoses encountered in Ireland. The use of menopausal hormone therapy is discussed as well as non-hormonal and non-pharmacological options.

**Conclusions:**

Managing menopausal symptoms is an important consideration for all physicians involved in the care of people living with and beyond a cancer diagnosis. High-quality data may not be available to guide treatment decisions, and, thus, it is essential to take into account the impact of the symptoms on quality of life as well as the likelihood of recurrence in each individual case.

## Introduction

Menopause, the final menstrual period, may be accompanied by a constellation of symptoms. Core menopause symptoms include vasomotor symptoms (hot flushes and night sweats) as well as urogenital symptoms such as vaginal dryness which may cause dyspareunia or discomfort with day-to-day living [1, 2]. There is a wide variation in the severity of these symptoms between women and in the same woman over time. Other symptoms associated with menopause may include sleep disturbance, mood disturbance, and muscle aches [3].

Menopause can also be induced by certain cancer treatments. Surgical removal of the ovaries in premenopausal women will cause premature or early menopause. Pelvic radiotherapy and certain forms of chemotherapy can also cause ovarian failure. Anti-endocrine therapy given for hormone sensitive malignancies can induce vasomotor symptoms. A diagnosis of cancer may also mean that previously effective menopause hormone therapy (MHT) is now contraindicated and, therefore, is discontinued leading to a resurgence in menopausal symptoms.

Evidence suggests that iatrogenic menopause may be more severe and long lasting than physiological menopause [4, 5]. Following a cancer diagnosis, women may be at an increased risk of affective disorders such as depression and anxiety, and menopausal symptoms such as sleep disturbance may exacerbate this risk. Furthermore, younger age at menopause may also be associated with psychological and sexual dysfunction and long-term health risks such as cardiovascular disease, osteoporosis and, potentially, cognitive dysfunction, and dementia [6].

Menopausal symptoms can be managed with hormonal, non-hormonal, and non-pharmacological therapies. MHT is an effective method of managing menopausal symptoms [7] but had been seen as contraindicated after some estrogen-sensitive cancers until more recently.

This document aims to summarise the available data to guide clinicians discussing management of menopause in the context of a cancer diagnosis. It is important to note that the use of MHT should take into consideration a woman’s symptom severity, impact on quality of life as well as her age and comorbidities, tumour stage, grade, and tissue of origin. All decisions should be multidisciplinary and patient focused, aiming to engage the woman in the management of her own symptoms and decisions regarding her care.

## Menopausal hormone therapy—non-oncological considerations for use

MHT can be given systemically in patch, gel, or oral forms. For women who have undergone hysterectomy estrogen only preparations should be used. For those with an intact uterus, estrogen and progesterone are recommended to protect against the risk of endometrial hyperplasia and cancer arising from unopposed estrogen. Multiple preparations are available. For local symptoms, vaginal estrogen can be effective, and, again, multiple preparations are available.

As a general rule, systemic MHT is best given via the transdermal route to minimise the risk of VTE. If progesterone is required, there is some data to suggest that micronised progesterone or dydrogesterone offers a more favourable side effect profile in terms of venous thromboembolic risk than the older forms of progesterone [8–11]. Micronised progesterone is a form of progestogen with an identical molecular structure to endogenous progestogen produced by the ovary [12]. The levonorgesterol intrauterine system (LNG-IUS, Mirena) is an adequate form of progesterone in those who require it or wish to avoid oral or transdermal progesterone.

The risk of venous thromboembolism (VTE) is elevated in the setting of malignancy. A personal or family history of VTE can be a complicating factor for people considering MHT in the context of a prior or current cancer diagnosis. When considering VTE risk, the route of administration is very important. Oral estrogen is associated with a two–fourfold increased risk of VTE. However, observational studies suggest that transdermal estrogen is not associated with increased VTE risk when compared to non-users [10, 13–15]. Again, micronised progesterone or dydrogesterone also offers a more favourable VTE risk profile when compared with other forms of progestogen [13–15]. The Mirena IUS is also acceptable in those who have an elevated VTE risk. If a patient is particularly high risk for VTE and is considering MHT, their case should be reviewed with a haematologist.

Duration of therapy is also an important discussion to have. For women with premature menopause, MHT should be offered until at least the natural age of menopause. The optimum dose and duration of MHT beyond this should be decided according to the severity of a woman’s symptoms as well as her response to therapy. Arbitrary limits should not be placed on duration of therapy [2].

## Tumour-specific considerations

### Endometrial adenocarcinoma

Endometrial adenocarcinoma is the most common gynaecological cancer diagnosed in Ireland with 557 people diagnosed in 2017 [16]. Although most commonly diagnosed in postmenopausal women, the incidence of endometrial cancer in premenopausal women has been increasing in recent years, likely directly related to increasing levels of obesity. Historical data suggests that less than 5% of endometrial cancer was diagnosed in women under the age of 40 [17]. However, more recent data suggests this proportion has risen as high as 18% [18]. Premenopausal endometrial cancer is associated with more favourable pathological features [19] as well as improved disease-specific survival when compared with older women [20]. The standard treatment for endometrial cancer is hysterectomy and bilateral salpingo-oophorectomy without or without pelvic lymph node dissection; however, oophorectomy can be avoided in women under 45 with early stage, low-grade tumours [21]. Given the favourable long-term prognosis for many younger women with endometrial cancer, managing menopausal symptoms in those who have their ovaries removed is likely to be an increasingly relevant area within survivorship care for this cohort of patients.

Unfortunately, there is a paucity of high-level data regarding the safe use of MHT in women after endometrial cancer. Only one randomised study has been conducted and, while it did not show an elevated risk of recurrence in those taking MHT; it was stopped early due to the initial findings of the Women’s Health Initiative study which raised safety concerns about the use of MHT. Therefore, it is difficult to draw conclusions from [22]. A Cochrane review of this and six other observational studies concluded that while there was no evidence of significant harm in early stage disease, there was a lack of high-quality evidence to guide the use of MHT and, indeed, no evidence at all in more advanced disease (FIGO stage II and beyond) [23].

Prevention of menopause symptoms in young women with endometrial cancer is a crucially undervalued intervention. Oophorectomy should be avoided in women under 45 if possible. Women in this age group need careful pre-operative molecular pathology work-up which should include mismatch repair immunohistochemistry to help identify those with Lynch Syndrome who may not be candidates for ovarian conservation due to their increased lifetime risk of ovarian cancer. In women who develop menopausal symptoms, the use of MHT after endometrial adenocarcinoma should be individualised, taking account of the severity of the woman’s symptoms, her preferences, and the uncertainty surrounding the use of MHT in this population. MHT should only be prescribed to address vasomotor symptoms.

### Uterine sarcoma

Uterine sarcoma is a rare form of gynaecological cancer which incorporates endometrial stromal sarcomas, carcinosarcomas (malignant mixed Mullerian tumours), and leiomyosarcomas. These types of cancer often express hormone receptors, and anti-endocrine therapy can be used in their treatment, and as such MHT is best avoided in this cohort of patients. Non-hormonal options should be utilised to manage troublesome menopausal symptoms, and these will be discussed later in this review.

### Epithelial cancer of the ovary, peritoneum, or fallopian tube

Epithelial ovarian cancer accounts for 90% of all ovarian cancers [24]. The majority (75%) of these are of the serous subtype with the remainder spread between the clear cell, endometrioid, and mucinous subtypes. Two meta-analyses have demonstrated that postoperative menopausal hormone replacement therapy for up to 4 years in patients with epithelial ovarian cancer does not increase the risk of cancer recurrence or reduce survival, consistent across grades and stages [25, 26]. Some of the observational data suggested a superior prognosis with MHT, echoed by a multinational randomised trial of 150 women published after these analyses, which demonstrated a trend towards improved overall and disease-free survival with MHT use, without any increase in adverse events (Table 1) [27].

**Table 1:** Summary overview

Cancer site	MHT use
Breast – hormone receptor positive	Avoid
Breast – hormone receptor negative	Individualise decision
Colorectal	Can be used
Lung	No consensus
Haematological	Can be used
Malignant melanoma	Appears safe in early-stage disease, avoid in advanced disease
Endometrial	Appears safe in early-stage disease, avoid in advanced disease
Uterine sarcoma	Avoid
Cervical	Can be used
Vulvar/vaginal	Can be used
*Epithelial ovarian*	
High-grade serous	Can be used
Low-grade serous	Avoid
Clear cell	Avoid
Endometrioid	Can be used
Mucinous	Can be used
Borderline	Can be used
*Non-epithelial ovarian*	
Sex cord stromal	Avoid
Germ cell	Can be used

It is worth noting, however, that numbers enrolled in these studies were low, many of them were not randomised and the majority of them was published more than 10 years ago. Despite these findings, there is still insufficient data to determine where MHT is safe in this population. We advise that decisions should be made based on histological subtype and severity of symptoms within a multidisciplinary team setting.

#### High-grade serous cancer

There is a lack of data specifically examining MHT use in high-grade serous tumours; however, they did make up the majority of cases included in the aforementioned meta-analyses and RCTs. Based on the available data, it is recommended that the use of MHT in this cohort be individualised based on severity of symptoms and the preferences of well-informed patients.

#### Low-grade serous cancer

Adjuvant, maintenance anti-endocrine therapy (tamoxifen or aromatase inhibitors) may be beneficial in low-grade serous carcinoma of the ovary. An observational study in 2017 showed improved overall and disease-free survival in women with stage II to IV low-grade serous carcinoma managed with maintenance anti-endocrine therapy following primary cytoreductive surgery and platinum-based chemotherapy (HR 0.44; 95% CI, 0.31 to 0.64; *p* < 0.001) [28]. Therefore, until sufficient safety data are available, clinicians are advised to avoid systemic MHT after low-grade serous epithelial ovarian cancer.

#### Non-serous ovarian cancers

A retrospective cohort study of 357 women with mucinous, endometrioid, and clear cell ovarian cancer demonstrated no significant difference in overall or disease-free survival with the use of MHT. There was also a non-significant trend for improved disease-free survival with MHT use in women under 55 years of age, regardless of FIGO stage and adjuvant therapy [29].

Therefore, systemic MHT can be considered in endometrioid and mucinous carcinoma of the ovary, but should be avoided in clear cell carcinoma as this is associated with increased risk of VTE [30].

Ultimately, the use of MHT in patients with ovarian cancer must be individualised, balancing the risk of recurrence and quality of life. There are many factors to consider including age at diagnosis and impact of symptoms as well as stage of disease. For many with a poor prognosis, maintaining a good quality of life should be prioritised.

#### Germ cell tumours

These tumours are relatively rare and most commonly affect younger girls and women between the ages of 10 and 30. They usually present with early disease and have an excellent prognosis. They are usually unilateral, and in general, surgical management is fertility sparing. There is no evidence to suggest that MHT should not be taken by this cohort of patients if required [31].

#### Sex cord stromal tumours

Granulosa cell tumours are the most common type of these tumours. They secrete hormones and often present with symptoms of hyperestrogenism. They may be treated with anti-endocrine treatment in some settings [32]. They have an indolent course, and patients may often experience late recurrences [33]. For these reasons, MHT is generally not recommended in this setting [31].

### Borderline ovarian tumours

Borderline ovarian tumours are more common among younger women and may account for up to one-third of ovarian malignancies diagnosed worldwide [34]. As with invasive ovarian tumours, several histological subtypes exist. The risk of recurrence depends on histological subtype [35]. There is a paucity of data about the use of MHT in this setting; however, it is considered reasonable to use in the setting of completely resected disease [31].

### Cervical cancer

Many new diagnoses of cervical cancer are in premenopausal women [36], and, therefore, the use of MHT should be considered. For many patients, ovarian conservation at the time of hysterectomy will avoid iatrogenic menopause; however, for patients with more advanced disease, for whom pelvic radiotherapy will be the primary form of treatment, menopause is inevitable. Surgical transposition of the ovaries out of the radiation field may preserve ovarian function and avoid menopause; however, long-term data to support his intervention remain sparse and are limited to single institutional cohorts [37]. Cervical cancers are most commonly squamous cell carcinomas with a smaller proportion being adenocarcinomas. Hormone receptor expression does vary between squamous and adenocarcinoma; however, this does not appear to affect survival [38].

There are few studies examining the use of MHT after cervical cancer treatment. One historical prospective study examined 120 patients with stage I or II cervical cancer, where eighty patients were given MHT and forty control patients were followed over a 5-year period. Patients receiving MHT demonstrated no significant difference in 5-year survival or cancer recurrence, but experienced improved quality of life with reduced menopausal symptoms and reduced complications of radiotherapy, suggesting benefits for MHT in these patients [39]. For the purposes of MHT use, cervical adenocarcinoma and squamous cell carcinoma should be treated the same.

It is important to remember that those with an intact uterus, even after radiotherapy do require combined MHT with estrogen and progesterone to avoid the risk of endometrial hyperplasia and malignancy.

### Vulvar and vaginal cancer

Vulval cancers represent approximately 5% of gynaecological cancers and are most commonly diagnosed in postmenopausal women. However, HPV-related vulval cancers are increasing in younger women. The majority of vulval cancers are squamous cell carcinomas and are not hormone dependent. Although data is limited, MHT use does not appear to alter prognosis in vulval cancer and can be used if required.

Paget’s disease of the vulva is a rare form of neoplasm in the vulval skin. It is most commonly diagnosed in postmenopausal women, and the primary treatment is usually surgery. MHT is best avoided in this cohort as extra-mammary Paget’s disease is associated with other malignancies and adenocarcinoma of the vulva [40].

Vaginal cancer is very rare, accounting for less than 1% of gynaecological malignancies. Similar to vulval cancer, most vaginal cancers are squamous cell carcinomas and are not hormone related. Given this, MHT is considered safe to use in the setting of vaginal cancer although it is not commonly required as most patients are diagnosed when postmenopausal.

### Colorectal cancer

Colorectal cancer is the third most common cancer in Ireland. Treatment can involve surgery, radiotherapy, and chemotherapy. In advanced disease, surgical menopause may be induced if it is necessary to remove the ovaries at the time of surgery. Pelvic radiotherapy is used in the neoadjuvant setting in rectal cancers and will induce menopause in female patients who are premenopausal. Colorectal cancer is increasingly diagnosed in those under 50 [41], so evidence-based management of menopausal symptoms is a growing issue in this population.

The use of combined MHT is associated with a lower risk of developing colorectal cancer [42]. Limited data suggest a benefit from taking MHT in terms of overall mortality and colorectal cancer-related mortality [43–46]. Overall, the existing evidence suggests that MHT can be safely prescribed, where necessary, to women with a history of colorectal cancer.

### Lung cancer

MHT does not appear to influence lung cancer incidence [42]. However, there are no studies examining disease-free survival or mortality associated with MHT in women with lung cancer [47] and, therefore, no specific guidelines exist. Some conflicting data regarding survival for women diagnosed with lung cancer and survival in those using MHT prior to the diagnosis do, however, invite caution in the use of MHT in this cohort of patients, and decisions should be made on case by case basis [47].

### Haematological malignancy

Haematological malignancies such as lymphoma and leukaemia sometimes require stem cell transplantation which results in premature or early ovarian failure in premenopausal women in 90% of cases [48]. MHT use in this situation is recommended and has not been shown to be associated with increased recurrence [48]. Consideration should be given to the preferential use of transdermal MHT in these cases due to the more favourable side effect profile in terms of the risk of venous thromboembolism in patients with haematological malignancies.

### Malignant melanoma

Little data exists around the safety of MHT after a diagnosis of malignant melanoma. A single study of 200 women with stage 1 or 2 melanoma suggests that MHT use does not alter prognosis [49]. Estrogen may in fact be protective in localised disease given the higher expression of ERβ in these tumours. ERβ is known to have suppressive effects on tumour proliferation, and increased expression of ERβ in melanoma is associated with improved prognosis [50]. No data exists for more advanced disease, and, thus, the decision to administer HRT should be made on an individualised basis.

### Breast cancer

The use of MHT after a diagnosis of estrogen receptor positive breast cancer is not recommended [51–54]. Several observational studies, cohort studies, and a systematic review seem to suggest that the use of MHT after breast cancer does not increase the risk of recurrence [55–60]. However, two independent randomised trials initiated in Sweden in 1997 to compare MHT with no MHT after diagnosis of early-stage breast cancer were terminated early in December 2003 due to safety concerns.

The HABITS trial [61] randomised more than 400 women to MHT (with or without progesterone in the form of norethisterone acetate) or no MHT. MHT was given for a mean duration of 2 years with follow-up for just over 4 years. HABITS was terminated early due to a higher risk of recurrence in the MHT arm (HR 3.3, CI 1.5–7.4). The Stockholm trial randomised 378 women to MHT (with or without medroxyprogesterone acetate) or no MHT. Duration of therapy was 2.1 years with median follow-up of 4.1 years [62]. It was terminated early by an independent data monitoring committee following the results of the HABITS trial; however, the Stockholm trial did not show a significant increase in recurrence (HR 0.81, CI 0.35–1.9). A joint analysis of the two trials showed that the risk of breast cancer recurrence was statistically significantly associated with MHT (HR = 1.8, 95% CI = 1.03 to 3.10), compared with no MHT [62]. A 10-year follow-up analysis of the Stockholm trial found that there was no difference in new breast cancer events. However, there was a higher rate of contralateral breast cancer in the MHT arm (HR 1.3, CI 0.9–1.9) [63]. It has been suggested that the differences in the use of progesterone between the two studies may have led to the difference in findings [64]. In any case, both studies were terminated prematurely, meaning that any results do not allow firm conclusions to be made. However, given these findings, ethical concerns mean that these studies will likely provide the best available evidence we will ever obtain on the use of MHT after breast cancer.

Tibolone is synthetic steroid medication with estrogenic properties. It is licenced for the management of VMS of menopause. The LIBERATE study from 2009 randomised over 3000 women with VMS on a background history of breast cancer to either tibolone 2.5 mg orally or placebo. They found a statistically significant higher rate of recurrence in the tibolone arm when compared with the placebo arm (HR1.40, CI 1.14–1.7) [65] meaning that tibolone is contraindicated in this population.

Non-hormonal management for vasomotor symptoms of menopause in women with a history of breast cancer should be considered a first-line treatment. NICE and the British Menopause Society (BMS) state that women with ongoing severe symptoms which are unresponsive to non-hormonal measures can consider the use of MHT in conjunction with their oncology care providers and menopause specialist, taking into consideration her own personal circumstances and tumour histological subtype [2, 51].

One third of women with breast cancer are diagnosed with either triple negative or HER2 positive, estrogen receptor negative breast cancer. For these women, it would seem intuitive that there would be no specific contraindication to the use of MHT in these patients. However, in reality, the use of MHT should be individualised given the limited and conflicting data on the use of MHT in this cohort of patients [61, 65].

### Non-hormonal pharmacological options for vasomotor symptoms

#### Selective serotonin reuptake inhibitors (SSRIs)/selective noradrenaline reuptake inhibitors (SNRIs)

Several classes of medication are used to manage vasomotor symptoms in those for whom MHT is contraindicated or declined. The most evidence exists for the use of SSRIs or SNRIs. These medications are traditionally used for depression and anxiety, but efficacy has been demonstrated for VMS. Commonly used medications include citalopram, fluoxetine, paroxetine, and venlafaxine. RCT level evidence supports a moderate benefit of these medications over placebo [66–71]. Two studies have compared venlafaxine to low-dose estradiol and found estradiol more effective than venlafaxine [72, 73].

**Fig. 1:**
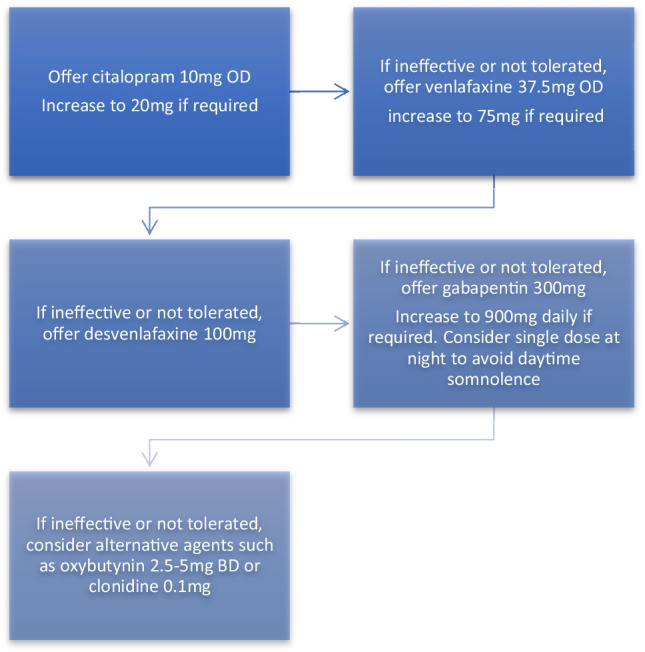
Suggested algorithm for management of troublesome vasomotor symptoms without the use of hormones. Adapted from Hickey et al. [83] An alternative approach is to discuss each of these medications and choose whichever is most acceptable to patients given expected side effects and patient preference

Overall, SSRI/SNRIs have a moderate effect on VMS but are less effective than standard dose MHT. Fluoxetine and paroxetine should not be used in patients taking tamoxifen due to theoretical concerns exist over an interaction between these medications which reduces the conversion of tamoxifen to its active metabolite [74]. Commonly recommended doses include citalopram 10–20 mg/day, venlafaxine 37.5 mg–150 mg/day, and paroxetine 10–15 mg/day (Fig. 1). Side effects include nausea, dry mouth, constipation, and sexual dysfunction.

#### Gabapentin

Gabapentin is traditionally used as an anti-convulsant or to manage chronic neuropathic pain [75]. Efficacy has also been demonstrated for VMS in RCTs [76, 77]. It is also recommended by international menopause societies for the management of VMS without hormones [52, 54, 78]. Side effects include somnolence and dizziness. It is recommended to start at a dose of 300 mg/day and gradually increase to 900 mg–2400 mg/day as tolerated or required. Gabapentin has also been shown to be inferior to standard dose MHT [79].

#### Other options

Other medications which have been shown to be of benefit are clonidine and oxybutynin. Clonidine is an alpha adrenergic agent which is used for hypertension and is licenced for management of VMS in some countries [80]. It is recommended that clonidine be started at a low dose of 25 μg twice daily and increased to 75–150 μg twice daily as required [52].

Oxybutynin is an anti-muscarinic and anti-cholinergic medication for the management of urinary urgency and urge incontinence. It has been shown to be superior to placebo for the management of VMS with similar efficacy rates as SSRI/SNRIs and gabapentin [81, 82]. Side effects include dry mouth, change in bowel habit, urinary tract infections, and nasopharyngitis. A dose of 2.5–5 mg twice daily is recommended.

### Management of urogenital symptoms

Urogenital symptoms of menopause include vaginal dryness, itching, and discomfort. These symptoms may cause sexual dysfunction but also discomfort with day-to-day living. Vaginal estrogen has been shown to be effective in managing these symptoms, although the level of evidence is low [84]. Women taking systemic MHT may also require vaginal estrogen for management of urogenital symptoms. The use of vaginal estrogen after hormone-sensitive cancers such as breast cancer is a much-debated topic. Observational data suggests no increased recurrence of breast cancer with the use of vaginal estrogen [59, 85–88]. However, systemic absorption of estrogen does occur at low levels from vaginal estrogen, but still within the general postmenopausal range. Nevertheless, this raises concerns for its use in women on aromatase inhibitors where the goal of therapy is to lower estrogen levels as much as possible [89, 90]. A dearth of data exists for gynaecological cancers [91]. Given the reassuring observational data in breast cancer survivors, it is reasonable to offer vaginal estrogen where required even in those for whom systemic MHT is not recommended in discussion with the treating oncologist.

In practice, non-hormonal vaginal lubricants and moisturisers are commonly used first line for urogenital symptoms in the setting of hormone-sensitive cancers despite uncertain evidence of benefit [92, 93]. Application of topical lidocaine prior to sexual intercourse has been shown to improve dyspareunia in breast cancer survivors [94]. Studies specifically in women with a history of breast cancer have shown superiority for silicone-based lubricant over water-based lubricant [95] as well as a benefit of olive oil as a lubricant in conjunction with vaginal moisturiser and pelvic floor relaxation techniques [96]. A recent study in endometrial cancer survivors showed a benefit in vulvovaginal health and sexual function with topical hyaluronic acid [97].

### Non-pharmacological options

Non-pharmacological therapies are used by up to 50% of women to manage menopausal symptoms, but many are not supported by high-quality evidence [98]. We will summarise here the non-pharmacological options which have evidence of benefit. They can be used alone or in conjunction with non-hormonal or hormonal medications. There is little high-quality evidence supporting exercise for the management of VMS [99, 100]. Higher BMI is associated with more severe and more frequent VMS [101, 102]. Available evidence seems to suggest that weight loss may reduce frequency and degree of bother or interference of VMS [103, 104].

Purpose designed cognitive behavioural therapy (CBT) for VMS has level 1 evidence to support its use with benefits seen in bother or interference of VMS as well as benefits on mood, sleep, and sexual function in menopausal women with and without a prior history of cancer [105–108]. This can be offered in person, via telephone, in groups or one-to-one. Availability of these programmes may be an issue; however, the British Menopause Society have a detailed factsheet which is freely available to women [109]. Hypnosis also has limited RCT level evidence to support its use in healthy postmenopausal women [110] and in those with a history of breast cancer [111] for the management of VMS with reduction in frequency of hot flushes as high as 68%. However, availability of hypnosis for VMS is likely to limit its usefulness in this setting.

### Specific populations

Women with a BRCA mutation who undergo risk reducing bilateral salpingo-oophorectomy at the recommended age are likely to experience surgical menopause [112]. For those women without a personal history of breast cancer, the available data suggests that MHT use for up to 4–5 years does not increase their rate of breast cancer [113, 114]. It is important to note that available evidence suggests that MHT does reduce but does not eliminate all symptoms [115].

For women with a history of Lynch syndrome who carry mutations in MLH1, MSH2, and MSH6, guidelines recommend risk reducing hysterectomy between the ages of 35 and 40 once childbearing is complete [116]. For those with a PMS2 mutation, oophorectomy can be omitted as their overall risk of ovarian cancer does not appear elevated. [117].

## Conclusion

Menopause after cancer can carry significant morbidity in terms of health and overall wellbeing and quality of life. Patients with hormone-sensitive cancers may pose a complex management challenge. Unfortunately, high-quality evidence to guide treatment may not be available, and, thus, each case must be considered individually, taking into consideration the risk of recurrence and the impact on activities of daily living. Discussions around the management of menopausal symptoms after a cancer diagnosis are an essential part of cancer management. To ensure the best outcomes for patients as they continue to navigate this difficult path, investigation of novel approaches to ameliorate symptoms is of tantamount importance. It is equally important that physicians remain knowledgeable of the most current data to ensure best patient outcomes.
